# Negative Effects of COVID‐19 in Hypertensive and Diabetic Patients: A Pre‐Post Study

**DOI:** 10.1002/hsr2.72335

**Published:** 2026-06-18

**Authors:** Soledad Gómez‐Escalonilla Lorenzo, Isabel Martínez, Blanca Notario Pacheco, Eva Rodríguez Gutiérrez

**Affiliations:** ^1^ University of Castilla‐La Mancha Cuenca Spain; ^2^ Health Service of Castilla‐La Mancha Castilla‐La Mancha Toledo Spain; ^3^ Department of Psychology University of Castilla‐La Mancha Cuenca Spain; ^4^ Faculty of Nursing Universidad de Castilla‐La Mancha Cuenca Spain; ^5^ Health and Social Research Center Universidad de Castilla‐La Mancha Cuenca Spain

**Keywords:** COVID‐19, diabetes mellitus, health‐related quality of life, hypertension, physical activity, treatment adherence

## Abstract

**Background and Aims:**

Restrictions during the COVID‐19 pandemic significantly affected the global population, particularly those with chronic conditions such as hypertension and diabetes, where lifestyle changes could have disrupted disease management. This study analyzes the association between reduced physical activity (PA) and variations in physical parameters, treatment adherence, and health‐related quality of life (HRQoL).

**Methods:**

A pre‐post study design was conducted from March 2019 to June 2021 in a Basic Health Area of central Spain. Clinical parameters (blood pressure, BMI, and HbA1c), PA levels, treatment adherence, and HRQoL (SF‐12) were measured in 152 patients before and after the lockdown. Analysis of covariance (ANCOVA) was used to compare mean differences across PA categories (quartiles), controlling for age and sex.

**Results:**

One year after the start of the lockdown, participants showed an increase in systolic blood pressure (*p* = 0.030) and significant decreases in PA (*p* = 0.004), treatment adherence (*p* < 0.001), and the mental component of HRQoL (*p* < 0.001). ANCOVA results indicated that the effect size (partial eta squared, *η_p_* ^2^) for differences in treatment adherence was 0.260, while for mental HRQoL, it was 0.040, suggesting a more moderate clinical relevance for the latter.

Subjects in the lowest physical activity category exhibited the poorest outcomes after adjusting for age and sex:

- Mental HRQoL difference: mean = −8.13 (95% CI: −11.11 to −5.15).
- Adherence difference: mean = −4.80 (95% CI: −5.94 to −3.65).

In contrast, participants with high PA demonstrated an improvement in treatment adherence (mean = 1.69; 95% CI: 0.31–3.06).

**Conclusions:**

Negative consequences of the pandemic remain observable 1 year after the lockdown in this population. Higher levels of physical activity are associated with mitigating some of these effects. However, due to the study design, caution is required when making direct causal interpretations.

## Summary

- The COVID‐19 pandemic has had a negative impact on physical activity, some clinical parameters, treatment adherence and mental HRQoL of diabetics and hypertensives in Spain.
- The negative effects of the pandemic on hypertensive and diabetic patients are observed 1 year after the onset of lockdown.
- Increased physical activity can mitigate some of these negative consequences. Those who are less physically active have poorer outcomes in adherence and physical and mental quality of life.

## 1 Introduction

On March 11, 2020, the World Health Organization (WHO) declared a global pandemic caused by severe acute respiratory syndrome coronavirus (SARS‐CoV‐2), which became the biggest international health emergency in recent years [1]. China, Italy, and Spain were among the most affected countries in terms of both the number of cases and deaths. Therefore, a strict lockdown was declared in these countries to prevent the spread of coronavirus disease (COVID‐ 19). In Spain, it was declared on March 17, cancelling all non‐essential services, travel, and the usual mobility of the population [2].

The COVID‐19 pandemic and the resulting lockdowns triggered radical changes in lifestyle habits, including significant reductions in physical activity (PA), altered dietary patterns, and increased psychological distress [3, 4, 5]. Multiple investigations have been carried out on this change of habits due to the COVID‐19 lockdown, the most frequent being inadequate eating habits [6], eating in response to stress or boredom [7], decreased PA [8], and increased alcohol consumption [9]. In addition, social isolation and other stressful circumstances, such as economic difficulties and loss of employment, have caused an increase in anxiety, stress, and sleep problems, which have affected the quality of life of the population [10]. Mental health has been seriously affected, with increasing levels of depression and anxiety [5, 10, 11].

These changes particularly affected individuals with non‐communicable chronic diseases, such as hypertension and diabetes, for whom lifestyle management is a cornerstone of treatment [12, 13]. Research conducted in Spain has begun to explore these impacts in primary care settings. For instance, a retrospective cohort study in Guadalajara involving 221 patients with hypertension and diabetes conducted 6 months after the lockdown showed that clinical parameters like HbA1c and BMI remained relatively stable [14]. Notably, that study found that telephone monitoring by nursing staff was essential in preventing clinical deterioration, as patients who received such follow‐up showed significant reductions in HbA1c and BMI compared to those who did not [14]. Another study in a rural Spanish population found no significant alterations in glycosylated hemoglobin but reported a perceived worsening of health control primarily attributed to sedentary lifestyles [15]. However, these studies face notable limitations, such as a focus on the immediate 6‐month post‐lockdown period and potential selection bias, as patients with the poorest disease control were often lost to follow‐up or avoided clinics due to fear of infection [14].

While initial studies documented the immediate negative impacts of confinement on blood pressure and glycemic management, the long‐term persistence of these effects remains a critical research gap. Current literature shows contradictory results regarding long‐term glycemic control, with some studies indicating deterioration and others showing improvement [16, 17]. Furthermore, while PA is known to influence both physical and mental health [18, 19], there is a lack of evidence regarding its specific role as a clinical buffer against the sustained effects of the pandemic 1 year after the initial lockdown in primary care settings [20, 21].

This study aims to clarify the association between PA levels and clinical and psychosocial outcomes in hypertensive and diabetic patients during this prolonged recovery period.

Based on the existing evidence, we formulated the following specific hypotheses:

1. A reduction in PA levels 1 year after the lockdown is positively associated with an increase in systolic blood pressure (SBP) and a worsening of glycemic control (HbA1c).
2. Lower levels of PA are linked to a significant decrease in treatment adherence and a decline in the mental component of health‐related quality of life (HRQoL).
3. Higher levels of post‐lockdown PA act as a buffer, maintaining better adherence and mental HRQoL compared to patients with low PA levels.

## 2 Methods

### 2.1 Study design

We implemented a longitudinal pre‐post methodology centered in a primary healthcare district in central Spain. The data collection period spanned from March 2019 to June 2021, allowing for a comparison of patient status before the pandemic and during the recovery phase.

The reporting of this study follows the STROBE (Strengthening the Reporting of Observational Studies in Epidemiology) statement guidelines for observational research to ensure a comprehensive and transparent description of the study processes.

### 2.2 Participants

The research cohort included adults (over 18 years of age) diagnosed with hypertension or diabetes who were registered within the local health system. Eligibility required a confirmed clinical diagnosis prior to the study and the cognitive capacity to provide informed consent. We excluded individuals who either declined to participate or were unable to communicate effectively in Spanish.

### 2.3 Sampling and Sample Size

Subjects were selected via a non‐probabilistic convenience sampling approach, with recruitment occurring through various nursing consultation rooms in the designated health area.

For a repeated measures design, with a significance level of 0.05 and sufficient statistical power (0.80), it is estimated that a sample of 100 participants would make it possible to detect a large effect size (*d* = 0.8) on the main outcomes.

A total of 159 participants completed the different scales after providing informed consent to participate in this study; 94 were hypertensive, and 65 were diabetic. A total of 159 questionnaires were collected before the COVID‐19 pandemic lockdown, and 152 were collected at least 1 year after the beginning of COVID‐19 lockdown (91 hypertensives and 61 diabetics).

### 2.4 Ethical considerations

The present study was approved by the Clinical Research Ethics Committee of the Area of Health from Toledo, Spain (Reg. 318), and all research was performed in accordance with the regulations of this committee. Study participants were all informed about the objectives of the study, and written informed consent was obtained prior to individual participation.

### 2.5 Instruments and Data Collection

The research utilized a structured 35‐item questionnaire divided into five specific domains to ensure multidimensional assessment:

- Demographics and medical history (6 items): including age, sex, marital status, education level, years since diagnosis, and comorbidities.
- Clinical parameters (4 items): physical parameters (SBP, DBP, BMI, and HB1Ac) were extracted directly from official medical records to avoid self‐reporting bias.
- Physical activity (1 item): assessed as a crude measure of frequency (days per week).
- Treatment adherence (12 items): to evaluate therapeutic compliance, we utilized the Spanish‐validated Martin–Bayarre–Grau (MBG) instrument [22]. This 12‐item scale assesses medication and lifestyle routines using a 5‐point Likert response format ranging from “never” (0) to “always” (4). Total scores (0–48) were used to categorize participants into levels of complete (48 to 38), partial (37 to 18), or non‐adherence (17 to 0).
  This questionnaire was validated in Spanish [22] and has been used in hypertensive populations [23]. Although it was initially validated for hypertension, it can also be adapted to other conditions such as chronic diseases or multimorbidity [24, 25].
  In our sample, the MBG questionnaire obtained a Cronbach's alpha of 0.889, indicating high internal consistency. For this study, Cronbach's alpha was calculated to assess the internal consistency reliability of the psychometric instruments. This metric indicates how closely related the set of items in a questionnaire is as a group; a value above 0.70 is generally considered acceptable for research purposes, signifying that the items consistently measure the intended construct.
  - Quality of life (12 items): measured using the SF‐12 health questionnaire, a 12‐item instrument derived from the more extensive SF‐36 [26]. This survey, originally designed by McHorney et al. [27] and validated in Spain by Alonso et al. [28], evaluates functional capacity and well‐being in individuals aged 14 and older. It provides two main summary metrics: a physical component score and a mental component score. Within this study's specific population of hypertensive and diabetic individuals, the questionnaire maintained internal consistency with a Cronbach's alpha of 0.76, confirming its reliability for this cohort.
  All heteroadministered questionnaires were completed anonymously following the clinical guidelines of the Clinical Research Ethics Committee of the Area of Health from Toledo, Spain. All participants (100%) completed all the items on the questionnaire. The source of clinical parameters was the patient's medical records.
  Data collection was divided into two distinct phases to assess the cohort's evolution:
  - Pre‐pandemic (baseline) period: data were collected from March 2019 through February 2020. This period established the participants' clinical and psychosocial status before any global health emergency was declared.
  - Post‐lockdown (follow‐up) period: data collection took place between March 2021 and June 2021. This timing was selected to ensure that at least one full year had elapsed since the start of the initial Spanish lockdown on March 17, 2020, allowing for an assessment of the long‐term persistence of clinical and psychosocial changes.

### 2.6 Plan of Analysis

Repeated measures analysis was carried out, using gender (men vs. women), age (< 65 vs. ≥ 65) and type of disease (hypertensives vs. diabetics) as intersubject factors, to check the differences between scores before and after the COVID‐19 pandemic lockdown in PA (days per week), treatment adherence, quality of life (physical and mental) and several clinical variables (systolic blood pressure, diastolic blood pressure, body mass index, and glycated haemoglobin).

Bivariate correlation coefficients were calculated to examine the relationship between PA and the other variables of the study: quality of life (physical and mental), treatment adherence, diastolic and systolic blood pressure, body mass index, and glycated haemoglobin. Those variables that correlated significantly with physical activity were included in an analysis of covariance (ANCOVA) to test the mean differences in the dependent variables by physical activity categories (model 0) as follows: low physical activity (first quartile), medium physical activity (second and third quartile) and high physical activity (fourth quartile); and controlling for age and sex (model 1).

Statistical significance was set at *p* < 0.05. Furthermore, to go beyond simple *p* values, we report partial eta squared (*η*_p_^2^) as a measure of effect size and 95% confidence intervals (CI) for all primary outcomes to indicate the precision and magnitude of the observed changes.

Sphericity was verified using Mauchly's test (*W* = 1.000, *p* > 0.05).

To maintain a family‐wise error rate and control for multiple comparisons, the Bonferroni correction was applied to all pairwise post‐hoc tests.

The analysis was based on a complete‐case approach; participants who did not complete both measurement points (due to death or transfer) were excluded, resulting in a final sample of 152.

All data analyses were performed using IBM SPSS Statistics (version 25.0).

## 3 Results

A total of 159 participants responded to the questionnaire before the pandemic lockdown, and 152 participants responded to the same questionnaire 1 year after the pandemic lockdown. This loss of participants was due to the death of 6 (3.8) and the transfer of one participant. A key finding in the follow‐up was the emergence of new‐onset conditions, specifically depression (7.2%) and anxiety (5.9%).

Table 1 shows the demographic and medical characteristics of the participants before and after the COVID‐19 pandemic lockdown.

**Table 1:** Demographic and medical characteristics.

Characteristics	Metric	Before the pandemic lockdown (*n* = 159)	One year after the pandemic lockdown (*n* = 152)
Age	mean ± SE	67.22 ± 12.53	67.01 ± 12.62
Sex	*n* (%)		
Male		78 (49.1)	72 (47.4)
Female		81 (50.9)	80 (52.6)
Marital status	*n* (%)		
Married		130 (81.8)	123 (80.9)
Unmarried		29 (18.23)	29 (19.07)
Education level	*n* (%)		
Tertiary		8 (5)	7 (4.6)
Secondary		23 (14.5)	22 (14.5)
Primary		88 (55.3)	85 (55.9)
Without studies		40 (25.15)	38 (25)
Chronic conditions			
Hypertensive patients	*n* (%)	94 (59.1)	91 (59.9)
Years of hypertension	mean ± SE	12.64 ± 9.9	12.39 ± 10.05
Diabetics	*n* (%)	65 (40.9)	61 (40.1)
Years of diabetes	mean ± SE	9.32 ± 6.27	9.26 ± 6.28
Comorbidities	*n* (%)		
Hyperlipemia		93 (58.5)	90 (59.2)
Obesity		68 (42.8)	63 (41.4)
Arthrosis		28 (17.6)	25 (16.4)
Depression		21 (13.2)	32 (21.05)
Tobacco use		14 (8.8)	13 (8.6)
Comorbidities after lockdown	*n* (%)		
Anxiety		—	9 (5.9)
Depression		—	11 (7.2)
Sleep disorders		—	6 (3.9)
Accidental falls		—	6 (3.9)
COVID‐19 infection	*n* (%)		
Yes		—	14 (9.2)
No		—	138 (90.8)

Abbreviation: SE, standard error.

Repeated measures analysis is shown in Table 2, comparing clinical variables (SBP, DBP, BMI, and HB1Ac), PA (days per week), adherence to treatment and HRQol (physical and mental component) before and after the COVID‐19 pandemic lockdown. A significant increase in SBP (*p* = 0.030), a significant decrease in physical activity (*p* = 0.004), in treatment adherence (*p* = < 0.001) and in the mental component of HRQoL (*p* = < 0.001) were observed.

**Table 2:** Repeated measures before and after the COVID‐19 pandemic lockdown, and with sex as an intersubject factor.

		Sex				
	Global *n* = 159			Men *n* = 78		Women *n* = 81		
Mean ± SE	Before *n* = 156	After *n* = 130	*p*	Before	After	Before	After	*p*
Clinical variables								
SBP	132.15 ± 11.98	135.87 ± 15.52	**0.030**	132.4 ± 9.65	134.48 ± 12.88	131.94 ± 13.64	136.99 ± 17.36	0.365
DBP	78.28 ± 6.47	78.58 ± 8.22	0.746	78.81 ± 6.61	78.14 ± 7.77	77.86 ± 6.37	78.94 ± 8.6	0.440
BMI	30.38 ± 5.01	30.31 ± 5.02	0.777	30.98 ± 5.19	30.64 ± 5.17	29.84 ± 4.82	30.02 ± 4.90	**0.035**
HB1Ac (diabetics *n* = 50)	6.79 ± 1.11	7.04 ± 1.09	0.062	6.63 ± 0.86	7.03 ± 1.06	6.76 ± 1.28	6.92 ± 1.10	0.432
*Physical activity*								
Days per week	3.98 ± 2.88	3.17 ± 3.01	**0.004**	3.86 ± 2.82	3.36 ± 2.98	4.1 ± 2.94	3.01 ± 3.04	0.188
Treatment adherence	35.83 ± 4.24	33.88 ± 3.53	**< 0.001**	36.03 ± 4.02	34.21 ± 3.53	35.65 ± 4.46	33.59 ± 3.53	0.390
*HRQoL*								
Physical component	41.17 ± 9.76	42.37 ± 8.19	0.101	42.90 ± 9.59	43.25 ± 7.76	39.60 ± 9.71	41.58 ± 8.53	0.299
Mental component	42.11 ± 9.3	37.1 ± 8.62	**< 0.001**	44.18 ± 8.63	40.73 ± 6.68	40.25 ± 9.54	33.83 ± 8.90	0.058

*Note:* Data are presented as mean ± standard error (SE). The values in bold indicate statistical significance at *p* < 0.05.

Abbreviations: BMI = body mass index, DBP = diastolic blood pressure, HB1Ac = glycated haemoglobin, HRQoL = health‐related quality of life, SBP = systolic blood pressure.

As intersubject factors we performed the analysis with sex, age, and type of disease; the results are presented in the Tables 2, 3, 4.

**Table 3:** Repeated measures before and after the COVID‐19 pandemic lockdown, and with age as an intersubject factor.

	Age				
	< 65 *n* = 59		≥ 65 *n* = 97		
	Before	After	Before	After	
Mean ± SE	*n* = 59	*n* = 46	*n* = 97	*n* = 84	*p*
Clinical variables					
SBP	128.30 ± 11.64	131 ± 15.92	134.25 ± 11.71	138.54 ± 14.71	0.702
DBP	80.20 ± 5.723	80.89 ± 8.63	77.24 ± 6.86	77.32 ± 7.75	0.626
BMI	31.77 ± 5.56	31.74 ± 5.53	29.50 ± 4.44	29.41 ± 4.47	0.655
HB1Ac (diabetics *n* = 50)	6.67 ± 1.07	7.19 ± 1.13	6.72 ± 1.12	6.83 ± 1.02	0.270
*Physical activity*					
Days per week	3.86 ± 2.74	2.76 ± 2.94	4.06 ± 2.98	3.44 ± 3.03	0.269
Treatment adherence	35.72 ± 3.83	33.38 ± 3.42	35.90 ± 4.51	34.21 ± 3.59	0.229
*HRQoL*					
Physical component	43.33 ± 10.87	45.31 ± 7.67	39.75 ± 8.75	40.45 ± 7.99	0.358
Mental component	41.65 ± 9.04	35.81 ± 9.01	42.42 ± 9.50	37.93 ± 8.30	0.318

*Note:* Data are presented as mean ± standard error (SE). The values in bold indicate statistical significance at *p* < 0.05.

Abbreviations: BMI = body mass index, DBP = diastolic blood pressure, HB1Ac = glycated haemoglobin, HRQoL = health‐related quality of life, SBP = systolic blood pressure.

**Table 4:** Repeated measures before and after the COVID‐19 pandemic lockdown, and with the type of disease as an intersubject factor.

	Type of disease				
	Hypertensives		Diabetics		
	*n* = 94		*n* = 65		
Mean ± SE	Before	After	Before	After	*p*
Clinical variables					
SBP	132.01 ± 10.33	136.92 ± 16.53	132.33 ± 14.03	134.44 ± 14.03	0.617
DBP	78.43 ± 6.92	79.07 ± 8.03	78.09 ± 5.85	77.93 ± 8.50	0.582
BMI	30.58 ± 4.92	30.45 ± 4.89	30.11 ± 5.17	30.13 ± 5.24	0.330
HB1Ac (diabetics *n* = 50)			6.79 ± 1.11	7.04 ± 1.09	0.062
*Physical activity*					
Days per week	4.16 ± 2.70	3.32 ± 3.04	3.72 ± 3.13	2.95 ± 2.96	0.777
Treatment					
Adherence	36.01 ± 4.39	34.19 ± 3.44	35.56 ± 4.03	33.43 ± 3.65	0.488
HRQoL	42.92 ± 8.88	43.82 ± 7.30	38.55 ± 10.48	40.20 ± 8.99	0.720

*Note:* Data are presented as mean ± standard error (SE). The values in bold indicate statistical significance at *p* < 0.05.

Abbreviations: BMI = body mass index, DBP = diastolic blood pressure, HB1Ac = glycated haemoglobin, HRQoL = health‐related quality of life, SBP = systolic blood pressure.

A significant interaction (*p* = 0.035) was found for body mass index; BMI decreased in men (30.98–30.64) but increased in women (29.84–30.02) during the pandemic year.

Mental HRQoL: While both sexes declined, the interaction was borderline (*p* = 0.058), with women showing a slightly more pronounced drop in mental well‐being scores (40.25–33.83) compared to men (44.18–40.73).

**Figure 1:**
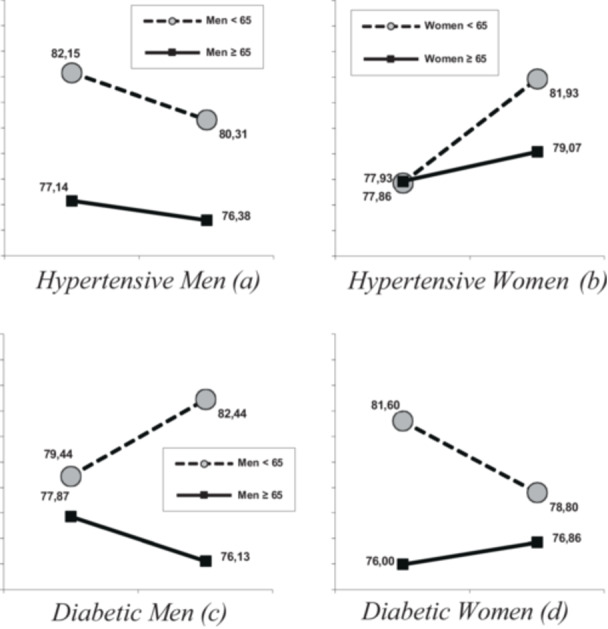
Interaction for sex, age and type of disease in DBP (a) hypertensive men, (b) hypertensive women, (c) diabetic men, and (d) diabetic women.

No differences were found by age or type of disease in the independent variables. However, an interaction of sex × age × type of disease was found in DBP (F(122, 1) = 122,000, *p*= 0.040). As can be noticed in Figure 1, the higher increase in DBP after COVID‐19 pandemic lockdown is observed in hypertensive women under 65 years old and in diabetic men both under 65 years old.

Bivariate correlation coefficients are presented in Table 5.

- PA correlations: physical activity showed positive and significant correlations with treatment adherence (*r* = 0.534), physical HRQoL (*r* = 0.277), mental HRQoL (*r* = 0.204), and diastolic blood pressure (DBP) (*r* = 0.262).
- Adherence and SBP: a significant correlation (*r* = 0.201) between adherence and systolic blood pressure suggests that patients who remained more adherent to their treatment also maintained better blood pressure control.

**Table 5:** Bivariate correlation coefficients (*r*) between physical activity, physical and mental HRQol, treatment adherence, diastolic and systolic blood pressure, body mass index, and glycated haemoglobin.

	Physical well‐being	Psychological well‐being	Treatment adherence	Diastolic blood pressure	Systolic blood pressure	Body mass index	Glycated haemoglobin
Physical activity	**0.277** **	**0.204** *	**0.534** **	**0.262** *	0.099	−0.138	−0.096
Physical HRQol		**−0.194** *	**0.220** *	−0.050	0.031	0.000	−0.030
Mental HRQol			0.073	0.115	−0.031	0.117	−0.018
Treatment adherence				0.147	**0.201** *	0.002	0.043
Diastolic blood pressure					**0.588** **	0.098	−0.064
Systolic blood pressure						0.134	−0.140
Body mass index							−0.229

*Note:* The values in bold indicate statistical significance.

* *p* < 0.05.

** *p* < 0.001.

The ANCOVA revealed that PA levels were significantly associated with differences in treatment adherence and mental HRQoL. However, the magnitude of these associations varied considerably. For treatment adherence, the effect size was large (*η*_p_^2 ^= 0.260), indicating that PA levels explain approximately 26% of the variance in adherence changes. In contrast, while the mental component of HRQoL showed statistical significance (*p* = 0.048), the effect size was small (*η*_p_^2 ^= 0.040), suggesting that its clinical relevance may be limited. Subjects with low PA (Q1) presented the greatest decline in both adherence (mean = −4.80, 95% CI [−5.94, −3.65]) and mental health (mean = −8.13, 95% CI [−11.11, −5.15]) (Table 6).

**Table 6:** Analysis of covariance (ANCOVA) for health outcomes by physical activity categories (adjusted for age and sex).

Variable	PA category	Adjusted mean difference (SE)	95% Confidence interval	*p* value	Effect size (*η*_p_^2^)
Mental HRQoL	Low (Q1)	−8.13 (1.51)	[−11.11, −5.15]	**0.048**	**0.040**
	Medium (Q2)	−3.67 (1.22)	[−6.09, −1.26]		
	High (Q3)	−3.51 (1.81)	[−7.09, 0.06]		
Treatment adherence	Low (Q1)	−4.80 (0.58)	[−5.94, −3.65]	**< 0.001**	**0.260**
	Medium (Q2)	−1.75 (0.47)	[−2.68, −0.83]		
	High (Q3)	1.69 (0.70)	[0.32, 3.06]		
DBP	Low (Q1)	−3.00 (1.21)	[−5.40, −0.60]*	**0.004**	—
	Medium (Q2)	1.73 (0.98)	[—]		
	High (Q3)	2.29 (1.52)	[—]		

*Note:* Data are presented as mean ± standard error (SE). Bold values indicate statistical significance at *p* value < 0.05.

## 4 Discussion

This study provides a specific assessment of the clinical and psychosocial status of hypertensive and diabetic patients 1 year after the initial COVID‐19 lockdown. While previous research documented the immediate negative impacts of confinement on lifestyle and mental health [3, 4], our findings suggest that these effects—specifically reduced PA, decreased treatment adherence, and lower mental health‐related quality of life (HRQoL)—have persisted well beyond the emergency phase [21].

### 4.1 Clinical and Psychosocial Persistence

The significant increase in systolic blood pressure and the decline in mental HRQoL align with documented global trends of pandemic‐related health deterioration [11, 19, 29]. However, our data highlight that these changes are not merely transient. One year post‐lockdown, participants remained less active and less adherent to treatments than in 2019 [30, 31, 32]. Notably, while the decline in mental HRQoL was statistically significant, the small effect size (*η*_p_^2 ^= 0.040) suggests that for many patients, the change may have limited clinical impact compared to the more robust decline seen in treatment adherence (*η*_p_^2 ^= 0.260).

### 4.2 The Role of Physical Activity

Our results show a significant association between post‐lockdown PA levels and health outcomes. Specifically, patients in the lowest PA category exhibited the most pronounced declines in treatment adherence and mental HRQoL. While these findings suggest a potential mitigating role for physical activity [18, 20, 33], we must avoid a definitive causal interpretation. The observed “buffer” effect likely reflects a complex interplay of behaviors; for instance, those who maintained higher PA levels may also have had better access to healthcare or fewer stressors [21, 34].

### 4.3 Implications for Clinical Practice

PA as a clinical marker: given the large effect size between physical activity and treatment adherence (*η*_p_^2^ = 0.260), PA levels should be assessed during routine visits as an indicator of adherence risk.

Protocols for monitoring patients with non‐communicable chronic diseases should incorporate periodic psychosocial assessments to address the long‐term mental health consequences of the pandemic.

### 4.4 Policy implications

From a public health perspective, these findings underscore the need for a shift in strategy:

- Given the importance of PA, health authorities should prioritize funding for community‐based PA programs. Such initiatives could foster systemic resilience, reducing the clinical burden on primary care during future health crises.
- Long‐term support frameworks: The persistence of health deterioration 1 year later indicates that policy responses must go beyond temporary emergency measures and move toward sustained longitudinal support for vulnerable populations with chronic diseases.

### 4.5 Study limitations

- Measurement validity: PA was assessed using a single, crude item, which lacks the precision of validated instruments.
- Confounding factors: the analysis controlled for age and sex, but unmeasured confounders—such as dietary changes or economic stress—could influence blood pressure and HRQoL [7, 10].
- Design constraints: the pre‐post design compares two specific time points but does not account for the natural progression of chronic diseases over 2 years.

## 5 Conclusions

The COVID‐19 pandemic has had a negative impact on physical activity, some clinical parameters, treatment adherence, and mental HRQoL of diabetics and hypertensives in Spain. On the other hand, post‐lockdown physical activity improves the physical parameters, adherence, and quality of life of these patients.

This research shows us that the adverse effects of the COVID‐19 lockdown are sustained over time, 1 year after the onset of the pandemic. And that increased physical activity can mitigate some of these negative consequences.

## Author Contributions

**Soledad Gómez‐Escalonilla Lorenzo:** conceptualization, investigation, writing – original draft, methodology, visualization, writing – review and editing, formal analysis, data curation, and resources. **Isabel Martínez:** conceptualization, investigation, funding acquisition, methodology, validation, visualization, writing – review and editing, software, formal analysis, project administration, data curation, supervision, and resources. **Blanca Notario Pacheco:** conceptualization, investigation, funding acquisition, writing – review and editing, visualization, validation, methodology, software, formal analysis, project administration, data curation, supervision, and resources. **Eva Rodríguez Gutiérrez:** formal analysis, software, project administration, data curation, validation, and visualisation.

## Funding

The authors have nothing to report.

## Conflicts of Interest

The authors declare no conflicts of interest.

## Transparency Statement

The corresponding author, Soledad Gómez‐Escalonilla Lorenzo, affirms that this manuscript is an honest, accurate, and transparent account of the study being reported; that no important aspects of the study have been omitted; and that any discrepancies from the study as planned (and, if relevant, registered) have been explained.

## Data Availability

Data that support the findings of this study are available from the corresponding author upon request.
